# Combined Phosphatase and Tensin Homolog (PTEN) Loss and Fatty Acid Synthase (FAS) Overexpression Worsens the Prognosis of Chinese Patients with Hepatocellular Carcinoma

**DOI:** 10.3390/ijms13089980

**Published:** 2012-08-10

**Authors:** Xuehua Zhu, Xia Qin, Maogui Fei, Wenmin Hou, Joel Greshock, Kurtis E. Bachman, Richard Wooster, Jiuhong Kang, Crystal Ying Qin

**Affiliations:** 1 Shanghai Key Laboratory of Signaling and Disease Research, School of Life Science and Technology, Tongji University, Shanghai 200092, China; E-Mail: huahuazxh@hotmail.com; 2 Department of Oncology, GlaxoSmithKline Research and Development Center, Shanghai 201203, China; E-Mail: xia.x.qin@gsk.com; 3 Cancer Research, GlaxoSmithKline, Collegeville, PA 19426, USA; E-Mails: joel.d.greshock@gsk.com (J.G.); kurtis.e.bachman@gsk.com (K.E.B.); richard.f.wooster@gsk.com (R.W.); 4 Institute for Nutritional Sciences, Shanghai Institutes for Biological Sciences, Graduate School of CAS, Chinese Academy of Sciences, Shanghai 200031, China; E-Mails: mgfei@sibs.ac.cn (M.F.); wmhou@sibs.ac.cn (W.H.)

**Keywords:** PTEN, FAS, prognosis, Chinese, hepatocellular carcinoma

## Abstract

We aimed to investigate the expression pattern of phosphatase and tensin homolog (PTEN), to evaluate the relationship between PTEN expression and clinicopathological characteristics, including fatty acid synthase (FAS) expression, and to determine the correlations of PTEN and FAS expression with survival in Chinese patients with hepatocellular carcinoma (HCC). The expression patterns of PTEN and FAS were determined using tissue microarrays and immunohistochemistry. The expression of PTEN was compared with the clinicopathological characteristics of HCC, including FAS expression. Receiver operator characteristic curves were used to calculate the clinical sensitivity and specificity of PTEN expression. Kaplan-Meier survival curves were constructed to evaluate the correlations of PTEN loss and FAS overexpression with overall survival. We found that the loss of PTEN expression occurred predominantly in the cytoplasm, while FAS was mainly localized to the cytoplasm. Cytoplasmic and total PTEN expression levels were significantly decreased in HCC compared with adjacent non-neoplastic tissue (both, *p* < 0.0001). Decreased cytoplasmic and total PTEN expression showed significant clinical sensitivity and specificity for HCC (both, *p* < 0.0001). Downregulation of PTEN in HCC relative to non-neoplastic tissue was significantly correlated with histological grade (*p* = 0.043 for histological grades I–II *versus* grade III). Loss of total PTEN was significantly correlated with FAS overexpression (*p* = 0.014). Loss of PTEN was also associated with poor prognosis of patients with poorly differentiated HCC (*p* = 0.049). Moreover, loss of PTEN combined with FAS overexpression was associated with significantly worse prognosis compared with other HCC cases (*p* = 0.011). Our data indicate that PTEN may serve as a potential diagnostic and prognostic marker of HCC. Upregulating PTEN expression and inhibiting FAS expression may offer a novel therapeutic approach for HCC.

## 1. Introduction

Hepatocellular carcinoma (HCC) is one of the most prevalent tumors worldwide [1], with very high incidences in China and Africa [2]. In China, HCC accounts for about 110,000 deaths annually [3]. Despite improvements in treatment modalities during the past few decades, the prognosis of HCC is still very poor because of frequent intrahepatic metastasis and tumor recurrence. Thus, further insight into the genes involved in hepatocarcinogenesis and novel therapeutic strategies is essential.

Phosphatase and tensin homolog (PTEN) [4] is a plasma membrane lipid phosphatase that acts as a tumor suppressor [5] and regulates several key cellular functions, including proliferation, apoptosis and migration [6]. PTEN dephosphorylates phosphatidylinositol triphosphate (PIP)-3 to PIP2, inhibiting the activation of the oncogene AKT [7], and then negatively regulates the phosphatidylinositol 3-kinase (PI3K)/Akt pathway. The PI3K/Akt pathway is important in terms of regulating growth, survival and proliferation of cells [7]. Studies of human cancer and mouse models suggest that alterations in PTEN, including mutation, loss of function, downregulation and loss of expression, might have some roles in pancreatic tumors, liver tumors, bladder tumors, adrenal pheochromocytomas, leukaemia and lymphoma [8]. Fatty acid synthase (FAS) is a key metabolic enzyme that catalyzes the synthesis of long-chain saturated fatty acids [9]. Overexpression of FAS occurs in many types of cancer and is frequently correlated with poor prognosis [10]. In prostate cancer cells, the loss of PTEN function plays an important role in the overexpression of FAS [10]. In mice, liver-specific deletion of PTEN results in a marked increase in liver FAS levels [11]. However, the correlation between PTEN loss and FAS overexpression in Chinese patients with HCC is still unclear and awaits further investigation.

In the present study, to gain further insights into the role of PTEN loss and FAS overexpression during HCC progression and prognosis, we determined PTEN expression and FAS expression in HCC tissues and paired adjacent non-neoplastic tissues collected from Chinese patients. We determined correlations between PTEN expression and clinicopathological characteristics, including FAS overexpression. We also determined correlations between PTEN and FAS expression with patient survival.

## 2. Results

### 2.1. Clinicopathological Characteristics of Patients with HCC

The clinicopathological characteristics of the 51 patients with newly diagnosed HCC are summarized in Table 1. There were 45 males with a mean age of 53.5 years (median, 54 years; range, 38–71 years; age was unknown in one male), and six females with a mean age of 56.7 years (median, 55.5 years; range, 48–72 years). In terms of histological stage, four patients were classified with grade I HCC, 34 with grade II HCC and 11 with grade III HCC. Histological stage was not determined in two patients. Regarding TNM (tumor-node-metastasis) stage, 38 patients were classified with stage I HCC, five with stage II HCC, six with stage III HCC and two with stage IV HCC. Depth of invasion was classified as T1 in 40 patients, T2 in five patients and T3 in six patients. Lymph node metastasis was classified as N0 in 50 patients and N1 in one patient. Distant metastasis was classified as M0 in 50 patients and M1 in one patient. The mean follow-up time was 30 months (median, 32 months; range, 0–60 months); 28 patients died (55%) during the follow-up, and the median follow-up of surviving patients was 48 months.

### 2.2. Loss of PTEN Expression in Patients with HCC

**Figure 1:**
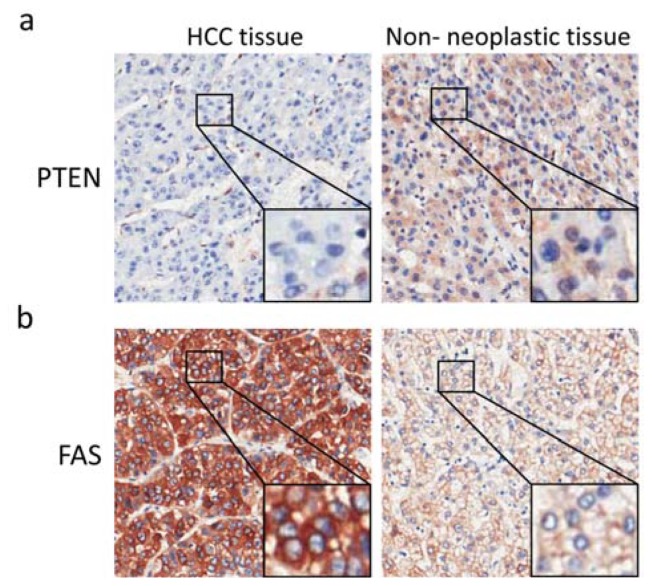
Representative images of PTEN (**a**) and fatty acid synthase (FAS) (**b**) expression in hepatocellular carcinoma tissue and paired adjacent non-neoplastic tissue.

Immunohistochemistry was performed to determine PTEN protein expression in HCC using a specific antibody for PTEN. Although PTEN was expressed in the cytoplasm and nucleus, it was mainly localized in cytoplasm of HCC and adjacent non-neoplastic tissues. Representative images of HCC and paired adjacent non-neoplastic tissues stained with PTEN antibody are shown in Figure 1a. Negative expression was defined as immunoreactive score (IRS) = 0, and positive expression was defined as IRS > 0. Overall, 25% of the tumor tissues were negative for total PTEN expression and 29% were negative for PTEN cytoplasmic expression (Table 1). By contrast, all of the adjacent non-neoplastic tissues were positive for total or cytoplasmic PTEN expression. The majority of HCC (84%) and adjacent non-neoplastic (92%) tissues were negative for nuclear PTEN expression (Table 1). The loss of cytoplasmic, nuclear or total PTEN expression was not significantly associated with any of the clinicopathological characteristics (Table 1).

### 2.3. Correlations between Downregulated PTEN Expression in HCC and Clinicopathological Characteristics

**Figure 2:**
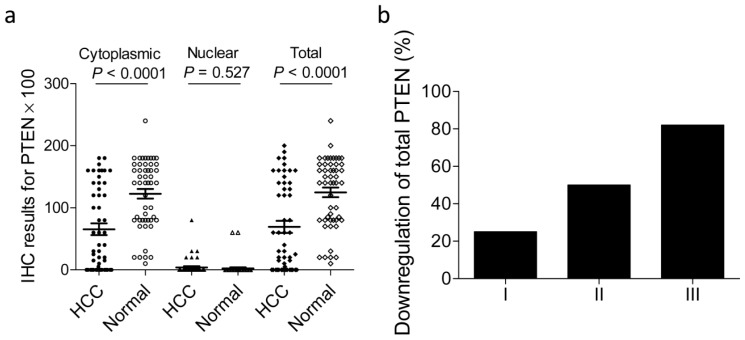
Correlation between downregulated PTEN expression in hepatocellular carcinoma and histological grade. (**a**) Scatterplot showing PTEN levels determined by immunohistochemistry in hepatocellular carcinoma and adjacent non-neoplastic tissues; (**b**) downregulation of PTEN expression was significantly correlated with histological grade (*p* = 0.043, comparing histological grade I–II *versus* III). HCC, hepatocellular carcinoma; Normal, adjacent non-neoplastic tissue; IHC, immunohistochemistry.

To investigate the effects of PTEN expression on the progression of HCC, we compared the expression of PTEN protein in HCC tissue with that in adjacent non-neoplastic tissue. We found that cytoplasmic and total PTEN expression was markedly decreased in HCC tissue than in non-neoplastic tissue (Figure 2a), indicating that PTEN expression is decreased in HCC. In addition, we compared the total PTEN level in HCC tissues with paired adjacent non-neoplastic tissues. We found that PTEN was downregulated in 57% HCC patients (Table 1). Further, we calculated the correlations between downregulated PTEN expression and the clinicopathological characteristics of patients with HCC. As shown in Figure 2b and Table 1, downregulated total PTEN expression was markedly correlated with histological grade of HCC (*p* = 0.043, for histological grades I–II *versus* grade III), but not with other clinicopathological characteristics.

### 2.4. Clinical Sensitivity and Specificity of PTEN Expression in HCC

**Figure 3:**
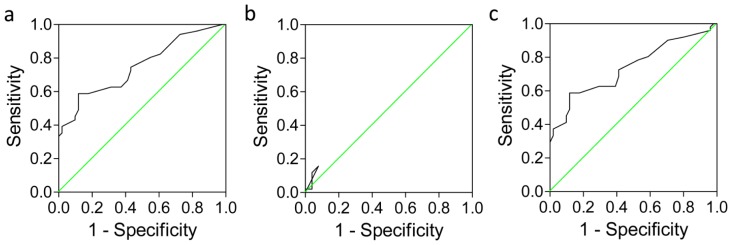
Clinical sensitivity and specificity of cytoplasmic (**a**); nuclear (**b**) and total (**c**) PTEN expression in hepatocellular carcinoma relative to adjacent non-neoplastic tissues. Receiver operator characteristic curves plot sensitivity *versus* 1–specificity. The area under the curve (AUC) represents the overall accuracy (range, 0–1.0). The green lines show an area of 0.5, which represents the accuracy achieved by chance alone. AUCs and optimal cut-off points are shown in Table 2.

We used receiver operator characteristic (ROC) curve analysis to delineate the clinical significance of PTEN expression in HCC. The area under the curve (AUC) represents the overall discrimination. The AUCs of cytoplasmic, nuclear and total PTEN expression in HCC were 0.752 (*p* < 0.0001), 0.539 (*p* = 0.494) and 0.739 (*p* < 0.0001), respectively (Figure 3a–c, Table 2). When using the optimal cut-off point determined by MedCalc software, the diagnostic accuracies of cytoplasmic, nuclear and total PTEN expression were 83.3%, 75.2% and 83.3%, respectively (Table 2). These data indicate that low cytoplasmic or total PTEN expression shows significant clinical sensitivity and specificity for HCC, and can differentiate HCC tissue from non-neoplastic tissue.

### 2.5. Correlation between PTEN Loss and FAS Overexpression in HCC

Previous studies have shown that liver-specific deletion of PTEN results in a marked increase in liver FAS levels in mice models [11]. Therefore, we determined FAS expression by TMA and immunohistochemistry in HCC and paired adjacent non-neoplastic tissue to evaluate the correlation between PTEN loss and FAS overexpression in HCC. Representative images showing FAS expression in HCC tissue and paired adjacent non-neoplastic tissue are shown in Figure 1b. FAS was exclusively expressed in the cytoplasm. By defining FAS overexpression as FAS IRS HCC/FAS IRS adjacent non-neoplastic tissue >1.5, we determined the correlation between loss of PTEN expression and FAS overexpression. As shown in Table 3, the loss of total PTEN expression was significantly correlated with FAS overexpression (*p* = 0.014), whereas the loss of cytoplasmic or nuclear PTEN expression was not associated with FAS overexpression.

### 2.6. Effects of PTEN Loss and FAS Overexpression on Survival of Patients with HCC

**Figure 4:**
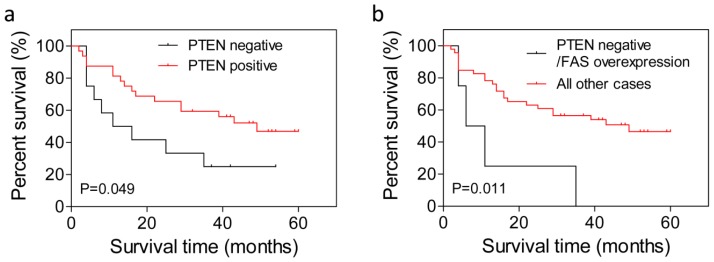
Impact of PTEN loss in combination with FAS overexpression on survival of patients with hepatocellular carcinoma (HCC). (**a**) Survival curve of patients with histological grades II–III HCC according to PTEN expression. The median survival time was 13.5 months *versus* 41 months in patients with PTEN-negative HCC *versus* patients with PTEN-positive HCC (*p* = 0.049); (**b**) Survival curve of patients with PTEN-negative and FAS-overexpressing HCC compared with all other types of HCC. The median survival time was 8.5 and 32 months in these two groups of patients, respectively (*p* = 0.011).

To evaluate the effects of loss of PTEN expression and FAS overexpression with prognosis of HCC, Kaplan-Meier survival curves were constructed. Our data revealed that, in patients with histological grades II–III HCC, cases negative for total PTEN expression had worse prognosis than cases positive for total PTEN expression (*p* = 0.049; Figure 4a). Among patients with histological grades II–III, the survival rate was 25% for cases negative for total PTEN expression *versus* 48% for cases positive for total PTEN expression. The median survival time of these two groups of patients was 13.5 and 41 months, respectively. When we considered PTEN loss in combination with FAS overexpression, HCC patients with PTEN loss plus FAS overexpression had significantly worse prognosis than other patients (*p* = 0.011; Figure 4b); survival rates were 0% and 51%, respectively, and median survival times were 8.5 and 32 months, respectively.

## 3. Experimental Section

### 3.1. Tissue Microarray

The HCC tissue microarray (TMA) used in the present study was from Shanghai Outdo Biotech Co. Ltd. This study was conducted according to the principles expressed in the Declaration of Helsinki. Human research ethics was approved by the Ethics Committee of the Third Xiangya Hospital, Central South University, Hunan, China. All patients gave written informed consent for participation in the study. In the TMA, 51 HCC tissues have paired adjacent non-neoplastic tissues. The 51 Chinese patients were newly diagnosed HCC, and none of the patients had received prior treatments for HCC, such as chemotherapy or radiotherapy. Clinicopathological data included sex, age at diagnosis, histological grade, TNM tumor stage, depth of invasion, lymph node metastasis, distant metastasis, and clinical follow-up data. All HCC tissues were histologically reviewed by one pathologist. Tumors were graded using the World Health Organization grading system. Patients were staged according to the TNM criteria proposed by the American Joint Committee on Cancer. Depth of invasion and lymph node metastasis were staged according to Union for International Cancer Control criteria. Survival time was defined as the time from the date of surgical diagnosis to the date of death or the date of last contact.

### 3.2. Immunohistochemistry and Scoring

Immunostaining of TMA slides was performed on a TechMate 500 automatic staining instrument (Dako A/S, Copenhagen, Denmark) according to the manufacturer’s instructions. Slides were incubated with PTEN (#9559, clone 138G6, Cell Signaling Technology, Beverly, MA, USA) or FAS (#3180, clone C20G5, Cell Signaling Technology, Danvers, MA, USA) monoclonal antibodies at a dilution of 1:50 overnight at 4 °C. Slides were incubated with a labeled polymer horseradish peroxidase detection kit (EnVision+; Dako, Carpinteria, CA, USA) for 30 min at 37 °C. Signal detection was done using a Dako signaling amplification system. A sample with previously confirmed expression of PTEN or FAS was used as a positive control. A negative control was established by replacing the PTEN or FAS antibody with control IgG (#3990, Cell Signaling Technology, Danvers, MA, USA). Protein expression was evaluated in terms of staining intensity and the percentage of positively stained cells. Staining was evaluated by one pathologist and two other observers simultaneously and independently, and a consensus was reached for each core. The staining intensity ranged from 0 to 3 (0 = negative, 1 = weak, 2 = moderate and 3 = strong). Immunohistochemical slides were semiquantitatively analyzed by determining the IRS [12], which was calculated as staining intensity × percentage of positive cells. Total protein (PTEN) expression was calculated as cytoplasmic protein expression + nuclear protein expression. Cases with total PTEN IRS of HCC tissue/total PTEN IRS of paired adjacent non-neoplastic tissue ≤0.5 were considered to show PTEN downregulation. Cases with FAS IRS of HCC tissue/FAS IRS of paired adjacent non-neoplastic tissue >1.5 were considered to show FAS overexpression.

### 3.3. Statistical Analysis

GraphPad Prism 5 (GraphPad software Inc., San Diego, CA, USA) and MedCalc software (MedCalc, Mariakerke, Belgium) were used for analyses. Correlations between PTEN expression and clinicopathological characteristics or FAS overexpression were analyzed using contingency tables and Pearson’s χ^2^ test or Fisher’s exact test, when appropriate. Student’s *t* test (two-tailed) was used to compare PTEN expression between HCC and adjacent non-neoplastic tissues. The clinical sensitivity and specificity of PTEN expression were determined using ROC curves. The Kaplan-Meier method was used to determine correlations between PTEN or FAS expression with survival time. Differences in survival times were analyzed using the log-rank test. In all of the tests, *p* < 0.05 was considered statistically significant.

## 4. Discussion

In the present study, we determined the expression patterns of PTEN and FAS in 51 HCC tissues and paired adjacent non-neoplastic tissues using TMA and immunohistochemistry. We found that the loss of PTEN mainly occurred in the cytoplasm in HCC tissues relative to adjacent non-neoplastic tissues, and that PTEN expression was markedly downregulated in HCC tissues compared with paired adjacent non-neoplastic tissues. Downregulation of PTEN expression was significantly associated with histological grade of HCC. The absence of total PTEN expression was significantly associated with FAS overexpression and with worse prognosis of patients with histological grades II–III HCC. Total PTEN negativity in combination with FAS overexpression was also associated with poor prognosis. Taken together, these data indicate that PTEN expression, or lack thereof, may be a useful diagnostic and prognostic marker in Chinese patients with HCC.

PTEN was originally discovered as a tumor suppressor gene in 1997. It is essential for regulating the PI3K/Akt signaling pathway [13], which is a key driver of cell growth, proliferation, metabolism, cell migration and cell invasion [14,15]. Deletion, mutation or other inactivation of PTEN is a common occurrence in the development and progression of many types of tumors [8]. The introduction of a wild-type PTEN gene into these cancer cells can inhibit cell growth, invasion and metastasis [8]. Therefore, PTEN plays an important role in tumor progression. Recent studies reported that PTEN localizes in both cytoplasm and nucleus, and cytoplasmic and nuclear PTEN play different roles [13,16,17]. In cytoplasm, PTEN can regulate cell growth by negatively regulating the PI3K/Akt signaling pathway [13]. In nucleus, PTEN controls genomic stability and cell cycle progression [16]. In our study, we found that cytoplasmic PTEN was lost in 29% of the HCC tissues, and total PTEN was lost in 25% of HCC tissues. By contrast, the majority of HCC (84%) and adjacent non-neoplastic (92%) tissues were negative for nuclear PTEN expression (Table 1). These data are similar to the findings in prostate cancer reported by McMenamin *et al.* [18] and Halvorsen *et al.* [19]. Genetic alterations may contribute to the reduced cytoplasmic PTEN staining [17,19]. PTEN loss in 29% cytoplasm of HCC tissues in our study may indicate the high incidence of PTEN mutation in the HCC. Since nuclear PTEN positively regulates DNA repair [16], our findings of PTEN loss in majority of the nuclei of both cancer and non-neoplastic tissues may suggest that the genomic DNA in these patients has been unstable and thus further promotes PTEN mutation and cytolasmic PTEN loss. However, the extract underlying mechanism needs to be further investigated. We also found that PTEN expression was markedly lower in HCC tissue than in the adjacent non-neoplastic tissue, consistent with the findings reported by Sze KM *et al.* [20] and Chen *et al*. [21]. The clinical sensitivity and specificity of low PTEN expression for HCC were also observed. In addition, our results showed that downregulation of PTEN was associated with poor differentiation, consistent with the findings reported by Tsung-Hui Hu *et al.* [22] and Shu-Kun *et al*. [23]. Overall, these results suggest that PTEN is involved in the development of HCC, and that PTEN downregulation is a marker for more aggressive HCC.

Previous studies have shown that PTEN regulates the expression of FAS *in vitro* [10] and *in vivo* [10,24] in a process mediated by the Akt signaling pathway. Overexpression of Akt-1 enhances FAS gene expression, while PTEN inhibits Akt-1 activation and thus negatively regulates the expression of FAS [9,10]. These observations indicate a functional relationship between PTEN and FAS. However, the relationship between PTEN expression and FAS expression has not, until now, been examined in clinical HCC tissues. In this study, we found that the loss of total PTEN was significantly correlated with FAS overexpression in HCC tissue.

HCC is well known to have a poor survival rate. Advanced disease usually accounts for the poor prognosis of HCC, irrespective of whether the patient receives surgery or chemotherapy. Although many studies have examined the prognostic impact of clinicopathological characteristics of HCC patients, these studies have limited clinical value. Tsung-Hui Hu *et al*. [22,25] reported that patients with reduced PTEN levels had shorter overall survival time compared with patients with normal PTEN expression. Our data revealed that the loss of PTEN was associated with poor prognosis, low survival rate and shorter survival time in patients with poorly differentiated HCC. Notably, patients with PTEN loss in combination with FAS overexpression had a worse prognosis than all of the other patients. From these results, we suggest that PTEN may serve as a molecular prognostic marker for poorly differentiated HCC, and that PTEN loss in combination with FAS overexpression is associated with even worse prognosis of HCC patients.

## 5. Conclusions

This study showed the expression patterns of PTEN and FAS in HCC. The loss of total PTEN was significantly correlated with FAS overexpression. PTEN downregulation was significantly associated with the histological grade of HCC. In patients with advanced HCC, PTEN loss was associated with poor prognosis, which was further worsened in combination with FAS overexpression. Taken together, PTEN could offer a potential diagnostic and prognostic marker of HCC, while overexpression of PTEN combined with inhibition of FAS may represent a novel anticancer therapeutic approach in HCC.

## Figures and Tables

**Table 1:** Clinicopathological characteristics of patients according to phosphatase and tensin homolog (PTEN) expression pattern.

Characteristic	Total Number	Cytoplasmic PTEN Negative	*p* Value	Nuclear PTEN Negative	*p* Value	Total PTEN Negative	*p* Value	Ca/CN Total ≤ 0.5	*p* Value
			
		15 (29%)		43 (84%)		13 (25%)		29 (57%)	
Sex									
M	45	13 (29%)	1	38 (84%)	1	11 (24%)	0.638	26 (58%)	1
F	6	2 (33%)		5 (83%)		2 (33%)		3 (50%)	
Age									
Median		52		55		52		54	
Range		43–72		38–72		43–72		39–72	
Histological grade									
I	4	1 (25%)	0.478	4 (100%)	0.404	1 (25%)	0.106	1 (25%)	0.043 a
II	34	9 (26%)		29 (85%)		7 (21%)		17 (50%)	
III	11	5 (45%)		8 (73%)		5 (45%)		9 (82%)	
TNM stage									
I	38	13 (34%)	0.336	33 (87%)	0.564	11 (29%)	0.419	22 (58%)	0.406
II	5	0 (0%)		4 (80%)		0 (0%)		3 (60%)	
III	6	2 (33%)		4 (67%)		2 (33%)		4 (67%)	
IV	2	0 (0%)		2 (100%)		0 (0%)		0 (0%)	
Depth of invasion									
T1	40	13 (33%)	0.315	35 (88%)	0.409	11 (28%)	0.37	22 (55%)	0.856
T2	5	0 (0%)		4 (80%)		0 (0%)		3 (60%)	
T3	6	2 (33%)		4 (67%)		2 (33%)		4 (67%)	
Lymph node metastasis									
N0	50	15 (30%)	0.515	42 (84%)	0.663	13 (26%)	0.555	29 (58%)	0.246
N1	1	0 (0%)		1 (100%)		0 (0%)		0 (0%)	
Distant metastasis									
M0	50	15 (30%)	0.515	42 (84%)	0.663	13 (26%)	0.555	29 (58%)	0.246
M1	1	0 (0%)		1 (100%)		0 (0%)		0 (0%)	

a Histological grades I–II *versus* grade III;

Ca, carcinoma tissue; CN, adjacent non-neoplastic tissue.

**Table 2:** Receiver operating characteristic curve analysis.

PTEN Expression Pattern	AUC (95% CI)	*p* Value	Optimal Cut-Off Point						

			Sensitivity	Specificity	+PV	−PV	+LR	−LR	DISEASE Accuracy
Cytoplasmic	0.752 (0.656–0.832)	<0.0001	58.8%	88.2%	83.3%	68.2%	5.00	0.47	83.3%
Nuclear	0.539 (0.438–0.638)	0.494	11.8%	96.1%	75.0%	52.1%	3.00	0.92	75.2%
Total	0.739 (0.642–0.821)	<0.0001	58.8%	88.2%	83.3%	68.2%	5.00	0.47	83.3%

The optimal cut-off point was defined by using the Medcalc software; AUC, area under the curve; CI, confidence interval; PV, predictive value; LR, likelihood ratio.

**Table 3:** Correlation between PTEN loss and FAS overexpression.

PTEN Expression Pattern	Total Number	FAS Overexpression	*p* Value
**Cytoplasmic PTEN**			
Negative	15	4 (26.7%)	0.054
Positive	36	2 (5.6%)	
**Nuclear PTEN**			
Negative	43	6 (14.0%)	0.572
Positive	8	0 (0.0%)	
**Total PTEN**			
Negative	13	4 (30.8%)	0.014
Positive	38	2 (5.3%)	
